# Prevalence and Characteristics of *Staphylococcus aureus* Isolated From Retail Raw Milk in Northern Xinjiang, China

**DOI:** 10.3389/fmicb.2021.705947

**Published:** 2021-08-09

**Authors:** Xiaomeng Kou, Huixue Cai, Shudi Huang, Yongqing Ni, Baolong Luo, Hao Qian, Hua Ji, Xingyi Wang

**Affiliations:** School of Food Science and Technology, Shihezi University, Shihezi, China

**Keywords:** *Staphylococcus aureus*, raw milk, classical enterotoxin, antibiotic resistance, biofilm, pulsed-field gel electrophoresis

## Abstract

*Staphylococcus aureus* is one of the main pathogens causing mastitis in dairy animals worldwide. It is an important opportunistic pathogen of raw milk, and the enterotoxin causes significant food poisoning. Monitoring the antibiotic resistance of *S. aureus* in raw milk is helpful for a risk assessment of *S. aureus*. In this study, 62 strains (43.1%) of *S. aureus* were isolated from 144 retail raw milk samples of different varieties from four regions in northern Xinjiang, China. Among them, the isolation rates at Shihezi, Hami, Altay, and Tacheng were 58.1% (54/93), 12.9% (4/31), 18.2% (2/11), and 22.2% (2/9), respectively. The isolation rate of positive strains in cow milk samples was the highest (61.7%, 37/60), followed by camel milk (35.9%, 23/64), and horse milk (10.0%, 2/20). The results of the classical virulence genes test showed that 12.9% (8/62) of the isolates carried at least one virulence gene. The main genotype was *see* (6.5%, 4/62), followed by *sea*+*sec* (3.2%, 2/62), *sea* (1.6%, 1/62), and *sec* (1.6%, 1/62). The analysis of 13 resistance genes and the susceptibility to 12 different antibiotics of 62 isolates showed that 80.6% (50/62) of the strains were resistant to at least one antibiotic, and 46.8% (29/62) were resistant to three or more antibiotics. The isolated strains had the highest resistance rate to penicillin (72.6%, 45/62), and 25.8% (16/62) of the isolates carried the *blaZ* resistance gene. In addition, 32 strains (51.6%, 32/62) of methicillin-resistant *S. aureus* were detected. All isolates had the ability to form biofilms. The pulsed-field gel electrophoresis results showed that the 47 isolates revealed 13 major pulsotypes (P1–P13) and 26 subtypes with 80% similarity, indicating the overall genetic diversity in the distribution area and sources of the samples. These findings indicate that *S. aureus* causes serious pollution of raw milk in northern Xinjiang, which has a negative effect on public health. Therefore, control measures and continuous monitoring should be undertaken to ensure the quality and safety of raw milk.

## Introduction

Dairy products are rich in protein, lactose, milk fat, and calcium, which are good sources of human nutrition. With the increasing interest of consumers in minimally processed foods, the consumption of raw milk has been gradually increasing (EFSA Panel on Biological Hazards, 2015). However, pathogenic microorganisms have been introduced into the dairy food chain (Song et al., 2015; Johler et al., 2018), and food-borne infections are reported frequently in China (Rong et al., 2017; Chen and Xie, 2019; Wu et al., 2019). Therefore, drinking raw milk may pose a risk to consumer health (Claeys et al., 2013).

*Staphylococcus aureus* is an important zoonotic pathogen, which can cause serious infection in humans and animals (Sergelidis and Angelidis, 2017; Papadopoulos et al., 2018). About 40.0% of mastitis cases in some countries are caused by *S. aureus* (Kateete et al., 2013; Basanisi et al., 2017). However, *S. aureus* may be discharged in the milk of dairy animals after infection, threatening consumer safety (Li et al., 2017). Therefore, bovine mastitis is a global challenge, as it not only damages the health of animals, but also reduces milk production and increases the cost of medical care, which eventually leads to huge economic losses in the dairy industry (Botaro et al., 2015).

*Staphylococcus aureus* has strong pathogenicity due to its wide distribution, high pollution rate, and fast transmission. It causes a variety of clinical manifestations, from mild-local, superficial skin lesions to serious invasive diseases, and may even threaten life (Turner et al., 2019). The symptoms and severity of infections caused by *S. aureus* are related to virulence factors (Xing et al., 2016). Among them, staphylococcal enterotoxins (SEs) are the major factor leading to foodborne poisoning. Food poisoning caused by *S. aureus* carrying SEs is very rapid in both occurrence and development, and poses great harm to human health (Basanisi et al., 2017). Among the previously discovered staphylococcal superantigen toxins, SEA, SEB, SEC, SED, and SEE are typical enterotoxins (Silva et al., 2015). Because of their high stability, these enterotoxins maintain activity in the digestive tract after ingestion (Fisher et al., 2018). According to reports, food poisoning caused by these five classical enterotoxins accounts for 95% of staphylococcal food poisoning (SFP) cases, while the remaining 5% of infections are related to newly discovered SEs (Korpysa-Dzirba and Osek, 2014; Papadopoulos et al., 2019).

Antibiotic treatment is an important measure to control bovine mastitis and human infection (Gomes and Henriques, 2016). *S. aureus* has significant resistance to antibiotics and the ability to evade the human immune system (Liu et al., 2017). More and more studies have reported that *S. aureus* has developed drug resistance and has evolved from single-drug resistant to multi-drug resistant (MDR), making it increasingly difficult to solve the problem of antibiotic resistance (Gomes and Henriques, 2016). Methicillin-resistant *S. aureus* (MRSA) represents those *S. aureus* strains that have acquired the *mecA* gene encoding penicillin-binding protein 2a, which mediates resistance to methicillin and all other β-lactam antibiotics, so it represents a global health problem (Arsic et al., 2012). Due to its seriousness and prevalence, nosocomial infection caused by MRSA has been listed as one of the three most difficult infectious diseases in the world by the World Health Organization (Becker and Wardenburg, 2015). Therefore, monitoring the antibiotic resistance of *S. aureus* in raw milk is very important for predicting the speed and type of antibiotic resistance development and for the decision-making of animal antibiotic treatment from the perspective of food safety (Liu et al., 2017).

Biofilm formation can enhance the virulence of bacteria, including *S. aureus*, and is considered one of the important virulence factors of *Staphylococcus* (Lee et al., 2014; Bissong and Ateba, 2020). The production of a biofilm not only enables bacteria to tolerate poor environments, but also reduces the penetration rate of antibiotics, promotes the horizontal spread of determinants of antibiotic resistance, and ultimately complicates the treatment of infections caused by these bacteria (Savage et al., 2013; Mathur et al., 2018).

*Staphylococcus aureus* is a primary pathogen of global public health concern and is ranked third in the world among reported foodborne pathogens (Umaru et al., 2016). Due to contamination of raw milk by pathogenic microorganisms, particularly the toxins produced by *S. aureus*, coupled with the increasingly serious drug resistance problem, research on the epidemic characteristics of *S. aureus* in raw milk has attracted widespread attention. Xinjiang is the most important dairy farming area in China. It has 3.6 million cows, with an average annual milk yield of 6,500 kg/cow (Dan et al., 2019). However, the raw milk is collected, processed, and transported in traditional ways in some areas of northern Xinjiang China, making it vulnerable to contamination by pathogenic microorganisms. Therefore, this study focused on the prevalence and characteristics of *S. aureus* in retail raw milk from different regions and sources in northern Xinjiang to help the relevant departments enact safety risk management measures for raw milk and dairy products, and provide a scientific theoretical basis for effective control of the spread of *S. aureus* in raw milk from the source to the table.

## Materials and Methods

### Sample Collection

A total of 144 samples of retail raw milk (including cow milk, camel milk, and horse milk) were collected at the farmers’ markets of four regions in northern Xinjiang during each quarter, including 93 samples from Shihezi, 31 samples from Hami, 11 samples from Altay, and 9 samples from Tacheng between August 2018 and October 2020. All raw milk samples were transferred to sterile bags and transported to the laboratory at 4°C for bacterial analysis.

### Isolation and Identification of *S. aureus*

The isolation and identification of *S. aureus* was referred from GB 4789.10-2016 “National Standard of the People’s Republic of China. Food Microbiological Examination: *S. aureus* National Food Safety Standard” (National Health and Family Planning Commission of the People’s Republic of China, and China Food and Drug Administration, 2016). The specific steps were as follows: 25 mL of prepared raw milk sample was added to 225 mL sterilized buffered peptone water, and incubated at 37°C for 24 h. Then, 3 mL of the raw milk bacterial culture was individually inoculated into a conical flask containing 30 mL of sterilized 7.5% sodium chloride tryptone soy broth (TSB) (Qingdao Hope Bio-Technology Co., Ltd., Qingdao, China) and incubated at 37°C for 24 h. Then, 100 μL of the turbid bacterial solution was spread on Baird-Parker (Qingdao Hope Bio-Technology Co., Ltd.) plates containing egg yolk potassium tellurite enrichment solution, and incubated at 37°C for 24 h. Several suspected colonies were picked and subcultured in brain heart infusion (BHI) (Qingdao Hope Bio-Technology Co., Ltd.) at 37°C for 24 h. Subsequently, the coagulase test was carried out by inoculating 0.8 mL of the bacterial suspension in BHI into ampules of freeze-dried rabbit plasma (Qingdao Hope Bio-Technology Co., Ltd.), followed by incubation at 37°C, and observation for 6 h. All isolates were stored at −80°C.

### Molecular Identification of *S. aureus* in Raw Milk by Polymerase Chain Reaction (PCR) Analysis

Genomic DNA was extracted using a Bacterial DNA Kit (Tiangen Biotech Co., Ltd., Beijing, China). Overnight *S. aureus* cultures in BHI were centrifuged (10,000 rpm, 1 min) using a labeled 2-mL Safe-lock tube. The pellet was collected and resuspended in 180 μL of digestion buffer (20 mM Tris, pH 8.0, 2 mM Na_2_-EDTA, 1.2% Triton, and 20 mg/mL lysozyme) obtained from Tiangen Biotech Co., Ltd., and incubated at 37°C for 30 min. Then, 20 μL of proteinase K and 220 μL of genomic lysis/binding buffer were added, and the mixture was shaken well for 15 s. The digestion mixture was incubated at 55°C for 30 min and 220 μL of absolute ethanol was added to each tube and mixed well. The solution and flocculent precipitate from each digestate were added to individual adsorption columns and centrifuged at 12,000 rpm for 30 s to remove the waste. Then, each column was washed twice by adding 500 μL of protein removal buffer followed by centrifugation at 12,000 rpm for 30 s to remove the waste. Each rinse solution was evaporated to dryness at 50°C. The DNA was eluted from the adsorption column by adding 100 μL of Tris-EDTA elution buffer followed by centrifugation at 12,000 rpm for 30 s. After quantifying and assessing the purity of the eluted DNA using a NanoDrop 2000c spectrophotometer (Thermo Fisher Scientific Inc., Waltham, MA, United States), the DNA was stored at −20°C for subsequent analyses.

Polymerase chain reaction (PCR) primers specific for the *S. aureus* thermonuclease gene (*nuc*) are shown in Table 1, and the PCR analysis was performed according to the methods described by Wang et al. (2012). The PCR amplification conditions were initial DNA denaturation at 94°C for 4 min, 37 cycles at 94°C for 60 s, 55°C for 30 s, 72°C for 90 s, and a final extension of 5 min at 72°C. The amplified PCR products were separated by 1.5% agarose gel electrophoresis and stained with Gold View DNA staining dye (Solarbio Science & Technology Co., Ltd., Beijing, China) at 100 V for 30 min. The PCR products were observed and recorded on a UV transillumination gel imaging system (Bio-Rad Laboratories Inc., Hercules, CA, United States).

**TABLE 1:** PCR primers for *nuc*, virulence, and antibiotic resistance genes.

Target gene	Nucleotide sequence (5′-3′)	PCR product size (bp)	Annealing temperature (°C)	References
*nuc*	GCGATTGATGGTGATACGGTT	279	55	Feng et al., 2016
	AGCCAAGCCTTGACGAACTAAAGC			
*blaZ*	TAAGAGATTTGCCTATGCTT	377	48	Feng et al., 2016
	TTAAAGTCTTACCGAAAGCAG			
*sea*	CTGTTCAGGAGTTGGATCTTC	156	60	Nagaraj et al., 2014
	CTTGAGCACCAAATAAATCG			
*seb*	CCAGATCCTAAACCAGATGAGTT	326	63	Nagaraj et al., 2014
	GTTTTTCGTTTATCAGTTTGATG			
*sec*	CCACTTTGATAATGGGAACTTAC	270	63	Nagaraj et al., 2014
	GATTGGTCAAACTTATCGCCTGG			
*sed*	CTAGTTTGGTAATATCTCCT	317	55	Grispoldi et al., 2019
	TAATGCTATATCTTATAGGG			
*see*	AGGTTTTTTCACAGGTCATCC	209	57	Grispoldi et al., 2019
	CTTTTTTTTCTTCGGTCAATC			
*ermA*	TCTAAAAAGCATGTAAAAGAA	645	45	Feng et al., 2016
	CTTCGATAGTTTATTAATATTAGT			
*ermB*	GAAAAGGTACTCAACCAAATA	639	45	Feng et al., 2016
	AGTAACGGTACTTAAATTGTTTAC			
*ermC*	TCAAAACATAATATAGATAAA	642	45	Feng et al., 2016
	GCTAATATTGTTTAAATCGTCAAT			
*aacA-aphD*	GAAGTACGCAGAAGAGA	491	45	Feng et al., 2016
	ACATGGCAAGCTCTAGGA			
*tetK*	GTAGCGACAATAGGTAATAGT	360	48	Feng et al., 2016
	GTAGTGACAATAAACCTCCTA			
*tetM*	AGTGGAGCGATTACAGAA	158	48	Feng et al., 2016
	CATATGTCCTGGCGTGTCTA			
*vanA*	GGGAAAACGACAATTGC	732	50	Feng et al., 2016
	GTACAATGCGGCCGTTA			
*rpoB*	AGTCTATCACACCTCAACAA	702	50	Feng et al., 2016
	TAATAGCCGCACCAGAATCA			
*linA*	GGTGGCTGGGGGGTAGATGTATTAACTGG	323	57	Dormanesh et al., 2015
	GCTTCTTTTGAAATACATGGTATTTTTCGA			
*optrA*	GCACCAGACCAATACGATACAA	794	55	Fan et al., 2016
	TCCTTCTTAACCTTCTCCTTCTCA			
*cfr*	TAAGAAGTAATAATGAGC	518	55	Fan et al., 2016
	TATAGAAAGTCTACGAGG			
*mecA*	GTGAAGATATACCAAGTGATT	147	46	Najarpeerayeh et al., 2014
	ATGCGCTATAGATTGAAAGGAT			

### Quantification of *S. aureus* in Samples by the Most Probable Number (MPN) Method

The quantification of *S. aureus* was performed according to GB 4789.10-2016 (National Health and Family Planning Commission of the People’s Republic of China, and China Food and Drug Administration, 2016) and the most probable number (MPN) method (Gombas et al., 2003). The 31 raw milk samples from Hami were not processed in time due to the long transportation distance. No quantitative monitoring of these samples was carried out to ensure the accuracy of the experimental data. The MPN of *S. aureus* in the sample was obtained from the MPN search table in GB 4789.10-2016 (National Health and Family Planning Commission of the People’s Republic of China, and China Food and Drug Administration, 2016).

### PCR Detection of Classical Virulence Genes

The PCR analysis of the purified DNA identified five classical virulence genes, including *sea*, *seb*, *sec*, *sed*, and *see*, present in isolated *S. aureus*. The PCR procedure was performed according to Nagaraj et al. (2014) and Grispoldi et al. (2019). The primers and the amplification conditions are shown in Table 1.

### Analysis of the Expression of Classical Virulence Genes by Enzyme-Linked Immunosorbent Assay (ELISA)

The Ridascreen^®^ Set A, B, C, D, E, enzyme-linked immunosorbent assay (ELISA) kit (R-Biopharm AG, Darmstadt, Germany) was used according to the manufacturer’s instructions to analyze the expression of virulence genes in *S. aureus* for at least one of the classical virulence genes (*sea*, *seb*, *sec*, *sed*, and *see*) (Carfora et al., 2015; Pexara et al., 2016). *S. aureus* isolates were cultured in BHI and incubated in a shaker at 37°C for 48 h. The cultures were centrifuged at 3,150 × *g* at room temperature for 15 min. A 100 μL aliquot of the supernatant was transferred to each well, and two negative controls and one positive control were set for each sample. After performing the ELISA, absorbance was measured at 620 nm, and the results were analyzed following the manufacturer’s instructions.

### PCR Amplification of the Antimicrobial Resistance Genes

The antimicrobial resistance genes were identified using purified bacterial DNA. All isolates were analyzed by amplifying various genes that confer resistance to antibiotics using simplex PCR, including penicillin (*blaZ*), methicillin (*mecA*), erythromycin (*ermA*, *ermB*, and *ermC*), gentamicin (*aacA-aphD*), tetracycline (*tetK* and *tetM*), vancomycin (*vanA*), rifampin (*rpoB*), clindamycin (*linA*), and linezolid (*optrA* and *cfr*). The resistance gene primers and PCR reaction conditions are shown in Table 1.

### Determination of Phenotypic Antimicrobial Resistance

Phenotypic antimicrobial resistance was determined by the broth microdilution method according to the Clinical and Laboratory Standards Institute (CLSI) (Clinical and Laboratory Standards Institute, 2020). The 12 antibiotics used in this study were erythromycin, clindamycin, penicillin, sulfamethoxazole/trimethoprim (19:1), ceftaroline, linezolid, tetracycline, vancomycin, gentamicin, ciprofloxacin, oxacillin, and rifampin. *S. aureus* isolates resistant to more than three classes of antibiotics were defined as MDR isolates (Magiorakos et al., 2012). *S. aureus* ATCC 25923 was used as the quality control strain.

### Biofilm Formation

The ability of the strain to form a biofilm was determined using the static microtiter plate method. The specific steps were referred from the method of Field et al. (2015) with minor modifications. Briefly, 62 strains were placed in test tubes containing TSB, incubated at 37°C for 24 h, and diluted to 10^8^ CFU/mL. Then, 2 μL of the diluted culture was added to 198 μL TSB (1:100) in a sterile 96-well culture plate. A 200 μL aliquot of TSB was used as the negative control. The biofilm was formed by 37°C incubation for 48 h. Then, the plate was removed and gently washed with 200 μL PBS. Subsequently, 200 μL of methanol was added to fix the formed biofilm for 15 min, and then 200 μL of 0.05% crystal violet (CV) was used to dye the biofilm for 15 min. The wells were washed three times in PBS to remove the excess dye. The microplate was air-dried and oscillated at 100 rpm for 30 min, and the biofilm combined with CV was dissolved in 200 μL of 33% (v/v) glacial acetic acid. The absorbance was measured using a microtiter plate reader (Bio-Tek Instruments Inc., Winooski, VT, United States) at 595 nm, and replicate experiments were carried out three times. The biofilm formation ability of the strains was divided into four types according to Stepanović et al. (2000): strong (+++, OD_595_ > 4 × OD_*C*_), medium (++, 2 × OD_*C*_ < OD_595_ ≤ 4 × OD_*C*_), weak (+, OD_*C*_ < OD_595_ ≤ 2 × OD_*C*_) and negative (−, OD_595_ ≤ OD_*C*_).

### Pulsed-Field Gel Electrophoresis (PFGE)

A selected subset of 47 isolates (41 from Shihezi, 2 from Tacheng, 2 from Altay, and 2 from Hami) recovered from 62 positive samples were analyzed by pulsed-field gel electrophoresis (PFGE) using *SmaI* as the restriction enzyme, according to a previous report (Szaluś-Jordanow et al., 2013). The electrophoresis (Bio-Rad CHEF Mapper XA system electrophoresis cell; Bio-Rad Laboratories, Hercules, CA, United States) running parameters were: initial pulse of 5 s, final pulse of 40 s, voltage of 6 V/cm, time of 18 h, and temperature of 14°C. The gels were stained with ethidium bromide for 30 min, washed in distilled water for 30 min, and photographed on a UV transilluminator gel imaging system (Bio-Rad Laboratories). The images were analyzed with BioNumerics 7.1 software (Applied Maths, Sint-Martens-Latem, Belgium) using dice coefficients and the unweighted-pair group method with arithmetic mean method to obtain the dendrograms. A cutoff value of 80% similarity was used to interpret the results. Isolates with an indistinguishable distribution for the same pulsotype and isolates with 80–99% similarity were designated as subtypes.

### Data Management and Analysis

In all cases, results that were below the minimum quantifiable level of 3 MPN/mL were set to 3, and MPN counts >1,100 MPN/mL were assigned the maximum value for this test (Stein and Tiefenthaler, 2005; Ji et al., 2011). The χ^2^ test and *t*-test were used to determine the prevalence and level of *S. aureus* in the raw milk samples from the four regions in northern Xinjiang. The associations between phenotypic and genotypic resistance were analyzed using the χ^2^ test. All statistical analyses were performed using SPSS 16.0 software (IBM Corp., Armonk, NY, United States). A *p*-value < 0.05 was considered significant.

## Results

### Prevalence and Levels of *S. aureus* in Raw Milk

A total of 62 *S. aureus*-positive strains (43.1%, 62/144) were isolated and identified from 144 retail raw milk samples (cow milk, camel milk, and horse milk) collected from four regions in northern Xinjiang (Shihezi, Hami, Altay, and Tacheng), and each positive strain originated from a different raw milk sample. Among them, 54 (58.1%, 54/93) *S. aureus* strains were isolated from 93 raw milk samples in Shihezi, 4 (12.9%, 4/31) *S. aureus* strains were isolated from 31 raw milk samples in Hami, 2 (18.2%, 2/11) *S. aureus* strains were isolated from 11 raw milk samples in Altay, and 2 (22.2%, 2/9) *S. aureus* strains were isolated from 9 raw milk samples in Tacheng. The isolation rate of *S. aureus* was highest in the cow milk samples, reaching 61.7% (37/60), followed by 35.9% (23/64) in camel milk, and 10.0% (2/20) in horse milk. The quantitative test results of the raw milk samples from all regions, except Hami, showed that the MPN pollution level at different sampling points ranged from 3 to 1,100 MPN/mL. The MPN positive range of the cow milk samples from Shihezi was the largest, and 9 cow milk samples were more than 1,100 MPN/mL (Table 2).

**TABLE 2:** Prevalence and levels of *S. aureus* at the different sampling sites.

Sampling site	Source of samples	No. of samples	No. (%) of *S. aureus* isolates	No. (%) of MRSA isolates	No. of samples with *S. aureus* densities in MPN/mL range						Positive range (MPN/mL)	*S. aureus* level (MPN/mL)^a^

					None detected	≤3	>3 to 10	≥10 to100	≥100 to 1100	≥1100		
Shihezi	Cow milk	59	36 (61.0%)	13 (36.1%)	23	4	4	16	3	9	0–1100	225.2
	Camel milk	34	18 (52.9%)	14 (77.8%)	16	4	4	7	3	0		
	Subtotal	93	54 (58.1%)	27 (50.0%)	39	8	8	23	6	9		
Hami	Camel milk	19	2 (10.5%)	0 (0%)	ND	ND	ND	ND	ND	ND	ND	ND
	Horse milk	12	2 (16.7%)	2 (100%)	ND	ND	ND	ND	ND	ND		
	Subtotal	31	4 (12.9%)	2 (50.0%)	ND	ND	ND	ND	ND	ND		
Altay	Camel milk	8	2 (25.0%)	2 (100%)	6	1	1	0	0	0	3–3.6	3.3
	Horse milk	3	0 (0%)	0 (%)	3	0	0	0	0	0		
	Subtotal	11	2 (18.2%)	2 (100%)	9	1	1	0	0	0		
Tacheng	Cow milk	1	1 (100%)	1 (100%)	0	0	0	1	0	0	9.2–23	10.1
	Camel milk	3	1 (33.3%)	0 (0%)	2	0	1	0	0	0		
	Horse milk	5	0 (0%)	0 (0%)	5	0	0	0	0	0		
	Subtotal	9	2 (22.2%)	1 (50.0%)	7	0	1	1	0	0		
Total		144	62 (43.1%)	32 (51.6%)	55	9	10	24	6	9	0–1100	210.1

*^*a*^The values are weighted averages shown as geometric means of positive samples; ND: no detection.*

### Detection of Classical SEs in Isolates by ELISA

**FIGURE 1:**
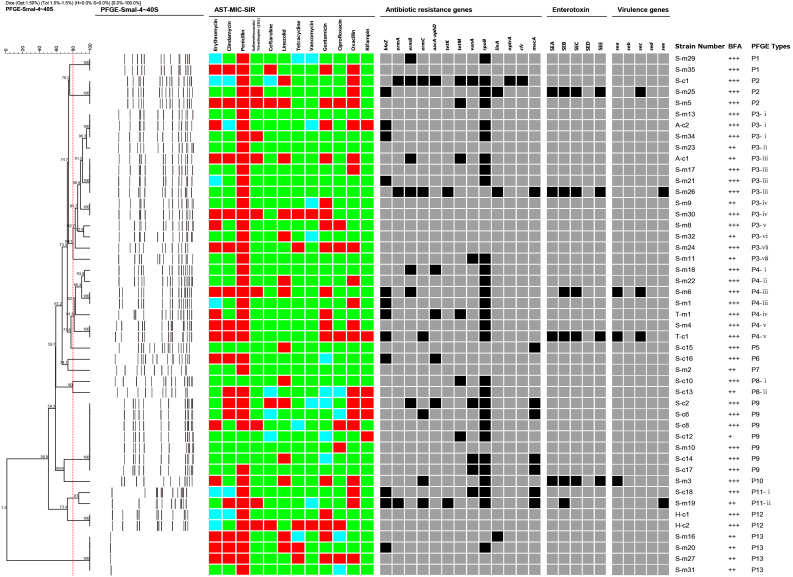
Dendrogram of PFGE patterns and antimicrobial susceptibility testing (AST), antibiotic resistance genes, virulence genes, enterotoxin production, biofilm forming and molecular characterization of 47 representative *S. aureus* strains isolated from raw milk in northern Xinjiang, China. The 47 isolates were grouped into 13 pulsotypes (P1–P13) by the PFGE patterns and all had more than 80% similarity. The AST results are shown as different colors according to the MIC values of the isolates to different antimicrobial agents. Green squares indicate susceptibility; blue squares indicate intermediate; and red squares indicate resistance. The detection of antibiotic resistance genes, virulence genes, and biofilm forming were summarized by a heat map. Black squares denote that the studied genes were detected in those isolates, or those isolates produced those types of enterotoxins. Gray squares denote that the isolates lacked the studied genes or could not produce those types of enterotoxins. BFA represents biofilm forming ability.

The ELISA analysis showed that all *S. aureus* isolates carrying virulence genes produced enterotoxin (Table 3 and Figure 1). Among them, each isolate produced SEB, and none of the isolates produced SED. In addition, the enterotoxin phenotypes of three isolates in milk samples from Shihezi and one isolate in camel milk from Tacheng were exactly the same, which could produce SEA, SEB, SEC, and SEE simultaneously.

**TABLE 3:** Toxin-gene profiles of enterotoxigenic *S. aureus* isolates from raw milk.

Strain number^a^	Virulence genes types	No. (%)	Enterotoxin production phenotype
S-m3	*sea*	1 (1.6%)	SEA, SEB, SEC, SEE
S-m6	*sea*+*sec*	2 (3.2%)	SEB, SEC
T-c1	*sea*+*sec*		SEA, SEB, SEC, SEE
S-m25	*sec*	1 (1.6%)	SEA, SEB, SEC, SEE
S-m19	*see*	4 (6.5%)	SEB
S-m26	*see*		SEA, SEB, SEC, SEE
H-h1	*see*		SEB
H-h2	*see*		SEB
Total		8 (12.9%)	

*^*a*^Strain number information: S: Shihezi; H: Hami; A: Altay; T: Tacheng; m: cow milk; c: camel milk; h: horse milk.*

### Detection of Classical Virulence Genes

Eight of 62 (12.9%) *S. aureus* strains carried at least one virulence gene (Table 3 and Figure 1). Among them, the main genotype was *see* (6.5%, 4/62), followed by *sea*+*sec* (3.2%, 2/62), *sea* (1.6%, 1/62), and *sec* (1.6%, 1/62). The *seb* and *sed* genes were not found in any of the positive isolates. In addition, among the eight isolates carrying virulence genes, five isolates were found in cow milk samples from Shihezi, two isolates in horse milk samples from Hami, and one isolate in camel milk samples from Tacheng.

### *S. aureus* Antibiotic Sensitivity

The sensitivity of the 62 *S. aureus* strains to the 12 antibiotics was tested by the broth microdilution method. The experimental range, the sensitivity breakpoints of the 12 antibiotics, the minimum inhibitory concentration distribution, and the positive antibiotic resistance results are shown in Tables 4, 5 and Figure 1. This results show that 80.6% (50/62) of the 62 isolates were resistant to at least 1 antibiotic, 46.8% (29/62) of the 62 isolates were resistant to 3 or more antibiotics. The penicillin-resistant strains accounted for the largest proportion (72.6%, 45/62), followed by oxacillin (37.1%, 23/62), and erythromycin (32.3%, 20/62). Notably, two strains of vancomycin-resistant *S. aureus* were found in cow milk samples from Shihezi and camel milk samples from Hami, respectively.

**TABLE 4:** Antimicrobial susceptibility of *S. aureus.*

Antimicrobial agent	Test range (mg/mL)	Distribution range (mg/mL)	MIC (mg/mL)		No. (%) of isolates		
				
			MIC_50_	MIC_90_	Resistant	Intermediate	Susceptible
Erythromycin	0.12–16	0.12–16	16	16	20 (32.3%)	7 (11.3%)	35 (56.5%)
Clindamycin	0.12–16	0.12–16	5	16	19 (30.6%)	4 (6.5%)	39 (62.9%)
Penicillin	0.06–8	0.06–8	6	8	45 (72.6%)	0 (0%)	17 (27.4%)
Sulfamethoxazole/Trimethoprim (19:1)	0.12–16	0.12–16	0.12	8	12 (19.4%)	0 (0%)	50 (80.6%)
Ceftaroline	0.25–32	0.25–16	0.5	1	5 (8.1%)	6 (9.7%)	51 (82.3%)
Linezolid	0.25–32	0.25–32	4	32	16 (25.8%)	0 (0%)	46 (74.2%)
Tetracycline	1–128	1–128	1	8	7 (11.3%)	4 (6.5%)	51 (82.3%)
Vancomycin	0.5–64	0.5–32	1	8	2 (3.2%)	8 (12.9%)	52 (83.9%)
Gentamicin	0.5–64	0.5–64	2	64	19 (30.6%)	6 (9.7%)	37 (59.7%)
Ciprofloxacin	0.25–32	0.25–32	0.5	16	10 (16.1%)	4 (6.5%)	48 (77.4%)
Oxacillin	0.5–32	0.5–32	4	32	23 (37.1%)	0 (0%)	39 (62.9%)
Rifampin	0.25–16	0.25–16	0.25	0.5	7 (11.3%)	0 (0%)	55 (88.7%)

*MIC Sensitivity standard according to Clinical and Laboratory Standards Institute (2020). The MIC values of sulfamethoxazole/trimethoprim (19:1) are given as trimethoprim MIC values.*

**TABLE 5:** Numbers and percentages of *S. aureus* isolates resistant to different antibiotics according to their region and source.

Antimicrobial agent	No. (%) of isolates											
	
	Shihezi			Hami			Altay		Tacheng			Total
	
	Cow milk	Camel milk	Subtotal	Camel milk	Horse milk	Subtotal	Camel milk	Subtotal	Cow milk	Camel milk	Subtotal	
Erythromycin	12 (19.4%)	2 (3.2%)	14 (22.6%)	0 (0%)	2 (3.2%)	2 (3.2%)	2 (3.2%)	2 (3.2%)	1 (1.6%)	1 (1.6%)	2 (3.2%)	20 (32.3%)
Clindamycin	11 (17.7%)	4 (6.5%)	15 (24.2%)	0 (0%)	2 (3.2%)	2 (3.2%)	1 (1.6%)	1 (1.6%)	1 (1.6%)	0 (0%)	1 (1.6%)	19 (30.6%)
Penicillin	29 (46.8%)	8 (12.9%)	37 (59.7%)	2 (3.2%)	2 (3.2%)	4 (6.5%)	2 (3.2%)	2 (3.2%)	1 (1.6%)	1 (1.6%)	2 (3.2%)	45 (72.6%)
Sulfamethoxazole/Trimethoprim (19:1)	7 (11.3%)	1 (1.6%)	8 (12.9%)	1 (1.6%)	2 (3.2%)	3 (4.8%)	1 (1.6%)	1 (1.6%)	0 (0%)	0 (0%)	0 (0%)	12 (19.4%)
Ceftaroline	2 (3.2%)	1 (1.6%)	3 (4.8%)	1 (1.6%)	1 (1.6%)	2 (3.2%)	0 (0%)	0 (0%)	0 (0%)	0 (0%)	0 (0%)	5 (8.1%)
Linezolid	8 (12.9%)	5 (8.1%)	13 (21.0%)	0 (0%)	2 (3.2%)	2 (3.2%)	1 (1.6%)	1 (1.6%)	0 (0%)	0 (0%)	0 (0%)	16 (25.8%)
Tetracycline	4 (6.5%)	0 (0%)	4 (6.5%)	1 (1.6%)	2 (3.2%)	3 (4.8%)	0 (0%)	0 (0%)	0 (0%)	0 (0%)	0 (0%)	7 (11.3%)
Vancomycin	1 (1.6%)	0 (0%)	1 (1.6%)	1 (1.6%)	0 (0%)	1 (1.6%)	0 (0%)	0 (0%)	0 (0%)	0 (0%)	0 (0%)	2 (3.2%)
Gentamicin	11 (17.7%)	0 (0%)	11 (17.7%)	2 (3.2%)	2 (3.2%)	4 (6.5%)	2 (3.2%)	2 (3.2%)	1 (1.6%)	1 (1.6%)	2 (3.2%)	19 (30.6%)
Ciprofloxacin	5 (8.1%)	0 (0%)	5 (8.1%)	1 (1.6%)	2 (3.2%)	3 (4.8%)	1 (1.6%)	1 (1.6%)	1 (1.6%)	0 (0%)	1 (1.6%)	10 (16.1%)
Oxacillin	12 (19.4%)	6 (9.7%)	18 (29.0%)	0 (0%)	2 (3.2%)	2 (3.2%)	2 (3.2%)	2 (3.2%)	1 (1.6%)	0 (0%)	1 (1.6%)	23 (37.1%)
Rifampin	0 (0%)	4 (6.5%)	4 (6.5%)	0 (0%)	1 (1.6%)	1 (1.6%)	1 (1.6%)	1 (1.6%)	1 (1.6%)	0 (0%)	1 (1.6%)	7 (11.3%)
Resistant to 1 antibiotic	10 (16.1%)	5 (8.1%)	15 (24.2%)	0 (0%)	0 (0%)	0 (0%)	0 (0%)	0 (0%)	0 (0%)	0 (0%)	0 (0%)	15 (24.2%)
Resistant to 2 antibiotics	5 (8.1%)	1 (1.6%)	6 (9.7%)	0 (0%)	0 (0%)	0 (0%)	0 (0%)	0 (0%)	0 (0%)	0 (0%)	0 (0%)	6 (9.7%)
Multidrug resistant	15 (24.2%)	6 (9.7%)	21 (33.9%)	2 (3.2%)	2 (3.2%)	4 (6.5%)	2 (3.2%)	2 (3.2%)	1 (1.6%)	1 (1.6%)	2 (3.2%)	29 (46.8%)

### Antibiotic Resistance Genes

The detection results of 13 antibiotic-resistant genes in the 62 isolates are shown in Table 6 and Figure 1. It showed that the proportion of isolates carrying *rpoB* gene was the highest (61.3%, 38/62), followed by *blaZ* (25.8%, 16/62), *mecA* (21.0%, 13/62), and *aacA-aphD* (19.4%, 12/62). Only one isolate carried *optrA* and *cfr* genes of linezolid. In addition, 6.5% (4/62) and 11.3% (7/62) of the isolates carried *tetK* and *tetM* genes, respectively. No isolates carrying *tetK*+*tetM* were found, but two isolates carrying *ermA*+*ermB*+*ermC* were found. Eleven isolates (17.7%, 11/62) carried the *vanA* gene, but the vancomycin phenotype of these strains was negative. Notably, 32 MRSA strains were detected in this study.

**TABLE 6:** Comparison of phenotypic and genotypic characteristics for antibiotic resistance in *S. aureus* isolates.

Antibiotics	Gene(s)	Characteristics of *S. aureus* isolates^*a*^						Association^*b*^
		
		G^+^	P^+^	P^+^/G^+^	P^–^/G^–^	P^+^/G^–^	P^–^/G^+^	*p*
							
		No. (%)	No. (%)	No.	No.	No.	No.	
Erythromycin	*Any*	15 (24.2%)	20 (32.3%)	4	31	16	11	0.755
	*ermA*	3 (4.8%)		0	39	20	3	
	*ermB*	11 (17.7%)		2	33	18	9	
	*ermC*	6 (9.7%)		2	38	18	4	
Clindamycin	*linA*	3 (4.8%)	19 (30.6%)	1	41	18	2	0.918
Penicillin	*blaZ*	16 (25.8%)	45 (72.6%)	12	13	33	4	0.801
Linezolid	Any	1 (1.6%)	16 (25.8%)	1	46	15	0	0.258
	*optrA*	1 (1.6%)		1	46	15	0	
	*cfr*	1 (1.6%)		1	46	15	0	
Tetracycline	Any	11 (17.7%)	7 (11.3%)	0	44	7	11	0.334
	*tetK*	4 (6.5%)		0	51	7	4	
	*tetM*	7 (11.3%)		0	48	7	7	
Vancomycin	*vanA*	11 (17.7%)	2 (3.2%)	0	49	2	11	0.504
Gentamicin	*aacA-aphD*	12 (19.4%)	19 (30.6%)	3	34	16	9	0.740
Oxacillin	*mecA*	13 (21.0%)	23 (37.1%)	4	30	19	9	0.751
Rifampin	*rpoB*	38 (61.3%)	7 (11.3%)	7	24	0	31	0.037

*^*a*^P^+^, phenotypic resistance; P^–^, phenotypic susceptibility; G^+^, resistance gene positive, G^–^, resistance gene negative.*

*^*b*^Association between resistance phenotypes and resistance genes.*

*A*p*-value < 0.05 was considered significant.*

In this study, the phenotypic resistance of rifampin was significantly correlated with genotype resistance (*p* < 0.05), while the other eight resistance rates were not correlated.

### Detection of Biofilm

The biofilm forming ability of all positive strains was tested using the static microtiter plate method, and the results are shown in Table 7 and Figure 1. Among the 62 isolates, 41 (66.1%, 41/62) isolates had strong biofilm forming ability, 20 (32.3%, 20/62) isolates had medium biofilm forming ability, and only 1 isolate (1.6%, 1/62) had weak biofilm forming ability. Among the cow milk samples, 25 isolates (40.3%, 25/62) had strong biofilm forming ability and 12 isolates (19.4%, 12/62) had medium biofilm forming ability; among the camel milk samples, 16 isolates (25.8%, 16/62) had strong biofilm forming ability, six isolates (9.7%, 6/62) had medium biofilm forming ability, and one isolate (1.6%, 1/62) had weak biofilm forming ability. The two isolates (3.2%, 2/62) from horse milk had medium biofilm formation ability.

**TABLE 7:** Biofilm forming ability of the *S. aureus* isolates.

Sampling site	Source of samples	No. (%) of isolates			
		
		Strong (+++)	Medium (++)	Weak (+)	Negative (−)
Shihezi	Cow milk	24 (38.7%)	12 (19.4%)	0 (0%)	0 (0%)
	Camel milk	12 (19.4%)	5 (8.1%)	1 (1.6%)	0 (0%)
	Subtotal	36 (58.1%)	17 (27.4%)	1 (1.6%)	0 (0%)
Hami	Camel milk	2 (3.2%)	0 (0%)	0 (0%)	0 (0%)
	Horse milk	0 (0%)	2 (3.2%)	0 (0%)	0 (0%)
	Subtotal	2 (3.2%)	2 (3.2%)	0 (0%)	0 (0%)
Altay	Camel milk	1 (1.6%)	1 (1.6%)	0 (0%)	0 (0%)
	Subtotal	1 (1.6%)	1 (1.6%)	0 (0%)	0 (0%)
Tacheng	Cow milk	1 (1.6%)	0 (0%)	0 (0%)	0 (0%)
	Camel milk	1 (1.6%)	0 (0%)	0 (0%)	0 (0%)
	Subtotal	2 (3.2%)	0 (0%)	0 (0%)	0 (0%)
Total		41 (66.1%)	20 (32.3%)	1 (1.6%)	0 (0%)

### PFGE Analysis

After the PFGE analysis of 47 *S. aureus* isolates, 13 pulsotypes (P1–P13) and 26 subtypes were obtained with 80% similarity (Figure 1). Among them, P3 (14/47), P4 (7/47), and P9 (7/47) were the main pulsotypes, followed by P13 (4/47) and P2 (3/47). P1, P8, P11, and P12 had two isolates (2/47), while P5, P6, P7, and P10 had only one isolate (1/47). The P9 group has 7 isolates with exactly the same type, which were respectively from camel milk and cow milk of Shihezi.

The PFGE clustering results revealed the overall genetic diversity in the distribution area and the sources of the samples. The isolates from Shihezi included 12 pulsotypes and 24 subtypes, and the isolates from Altay and Tacheng included one pulsotype and two subtypes. The isolates from cow milk samples included nine pulsotypes and 20 subtypes, and the isolates from the camel milk samples included nine pulsotypes and 11 subtypes.

## Discussion

In this study, the *S. aureus* isolation results of the 144 raw milk samples collected from different milk sources in four regions of northern Xinjiang showed that the total isolation rate of positive strains was 43.1%, and the pollution level was high, which was consistent with the results of Traversa et al. (2015) and Ahmed et al. (2019). Liu et al. (2017) determined that the isolation rate of *S. aureus* was 27.7% in 195 raw milk samples collected from northern China. Zhao et al. (2020) studied bulk tank milk (BTM) samples from dairy farms in Shandong Province, and found that the *S. aureus* contamination rate was 28.9%, which was slightly lower than this study. *S. aureus* contaminates many sources of raw milk, which are usually related to mastitis or human carriers. Failure to follow good animal husbandry and food processing methods will lead to pollution of the finished product (Schmidt et al., 2017). In conclusion, this difference is largely due to differences in the type of livestock breeding system, animal species, milking method, and the surrounding sanitary conditions (Umaru et al., 2016). Therefore, the collection, production, transportation, and sale of raw milk should be standardized. At the same time, it is necessary to carry out corresponding professional training for the operators of each link to minimize the pollution of raw milk caused by adverse conditions and to avoid further harm to consumer health.

In this study, 12.9% (8/62) of the *S. aureus* strains carried one or more virulence genes, and four different genotypes were present. Among them, 6.5% (4/62) of the strains had the *see* genotype, and 3.2% (2/62) of the strains had the *sea*+*sec* genotype. The remainder belonged to the *sea* (1.6%, 1/62) and *sec* genotypes (1.6%, 1/62). The *sea* and *sec* genes have been detected in relevant studies that determined the *S. aureus* toxin genes in raw milk, which is consistent with the results of this study (Cavicchioli et al., 2015; Xing et al., 2016). None of the strains detected here carried the *seb* or *sed* genes. Pexara et al. (2016) reported that no *S. aureus* isolates in caprine milk carry the *seb* gene. Obaidat et al. (2018) also did not detect the *seb* gene and only 0.6% of the *sed* gene was detected in Jordan’s BTM. Different studies have reported different prevalence rates of the virulence genes, which may be attributed to geographical distribution and the sources of the samples (Zhao et al., 2020). In this study, all isolates carrying virulence genes detected by ELISA produces at least one enterotoxin. Among them, SEB and SEC had the higher production frequency, which was consistent with the results of Carfora et al. (2015) and Pexara et al. (2016). Therefore, milk and dairy products are important reservoirs of enterotoxin-producing *S. aureus*. This result also reveals the potential threat to raw milk, and indicate that it is necessary to implement a monitoring program and preventive measures. Additionally, education and promotion of the health of livestock farmers and consumers should be carried out in the community to improve the prevention and vigilance of SFP caused by poor production and eating methods.

Antibiotic therapy is an important method to control mastitis, but the effect of antibiotic therapy on *S. aureus* is deteriorating with the worldwide acceleration of antibiotic resistance (Gomes and Henriques, 2016). Therefore, antibiotic use should be monitored to predict the development speed and type of antibiotic resistance, and can also be used to make decisions on animal antibiotic treatment from the perspective of food safety, which is helpful when evaluating the risk of *S. aureus*.

The sensitivity test of the 12 antibiotics by the broth microdilution method showed that the *S. aureus* isolates had the highest resistance rate to penicillin (72.6%, 45/62), and 25.8% (16/62) of the isolates carried the *blaZ* resistance gene. This high frequency was consistent with the findings of previous studies (Jamali et al., 2014; Feng et al., 2016; Qu et al., 2019), mainly because β-lactam is widely used in the treatment of bovine mastitis in China and Turkey (Aslantaş and Demir, 2016). Additionally, penicillin is the first choice to treat *S. aureus* in human medicine (Thongratsakul et al., 2020). However, it is reported that penicillin resistance is increasing in some foreign countries (Jahan et al., 2015; Aslantaş and Demir, 2016). The drug resistance of *S. aureus* varies from region to region and is affected by the use of antimicrobial agents (Ren et al., 2020). In China, the resistance rates of penicillin are 94.3% in Ningxia (Wang et al., 2016), 80.5% in Shaanxi (Li et al., 2015), 74.4% in Shandong (Zhao et al., 2020), and 31.3% in Beijing (Wang et al., 2018). Based on these high resistance rates, penicillin should be used cautiously for mastitis caused by *S. aureus*.

In this study, no significant correlations were detected between the genotypes and phenotypes of most of the strains in terms of virulence or drug resistance, which was similar to some studies. This result may have occurred because phenotypic presentation is determined by many factors, such as point mutation, biofilm formation, and antibiotic tolerance, and genotype is only determined by one of the internal factors (Bissong and Ateba, 2020).

*Staphylococcus aureus* is generally resistant to multiple antibiotics and is MDR, which has a strong effect on the dairy industry and food public safety (McDougall et al., 2014). In this study, 50 (80.6%, 50/62) strains of *S. aureus* were resistant to at least one antibiotic, which is close to some domestic research reports (Liu et al., 2017; Ren et al., 2020), but much higher than some foreign countries, such as Italy (39.4%) and Poland (28.3%) (Rola et al., 2016; Giacinti et al., 2017). Notably, the proportion of MDR in this study was high (46.8%, 29/62), so the pathogenicity and transmission risk of *S. aureus* cannot be ignored. The control of strains that express the MDR phenotype and have the ability to produce biofilms and enterotoxins remains an important public health issue (Cavicchioli et al., 2015; Wang et al., 2016, 2018).

*Staphylococcus aureus* is considered to be the main cause of hospital and community-acquired infections (Gopal and Divya, 2017). Thirty-two (51.6%, 32/62) MRSA strains were found in this study, which was close to the report of Al-Ashmawy et al. (2016) (53.0%), but higher than the report of Aqib et al. (2017) (34.0%). In addition, 50.0% (16/32) of the MRSA were isolated from camel milk samples collected in Shihezi and Altay, 43.8% (14/32) were isolated from cow milk samples in Shihezi and Tacheng, and the remaining 6.3% (2/32) were collected from horse milk samples in Hami. Among the 32 MRSA, 20 strains were MDR. Many food poisoning outbreaks associated with *S. aureus* are caused by MDR *S. aureus*, including MRSA (Johler et al., 2015; Jans et al., 2017).

The drug resistance of *S. aureus* increases gradually due to the evolution of the bacterium and the abuse of antibiotics. The MRSA infection rate is increasing worldwide, and MRSA has become an important bacterial pathogen in hospitals (Song et al., 2016; Tenhagen et al., 2018). The *S. aureus* resistance mechanism is complex, particularly that of MRSA. Therefore, it is of great guiding significance to understand the drug resistance of *S. aureus* in time and study the drug resistance characteristics and mechanisms of *S. aureus* for the use of antibiotics in animal husbandry.

The microtiter plate is one of the most commonly used techniques to quantify biofilm forming ability (Angelopoulou et al., 2020). Many studies comparing the sensitivity of phenotypic biofilm production have reported that the microtiter plate assay is more sensitive, specific, and accurate than Congo Red Agar (Torlak et al., 2017; Triveni et al., 2018). This study found that all isolates had the ability to form a biofilm, which is consistent with the study of Wang et al. (2018). Bissong and Ateba (2020) also reported that 90.9% (70/77) of isolates are biofilm producers. In addition, we found that most isolates (66.1%, 41/62) were strong biofilm-forming strains. However, the study of Khasawneh et al. (2020) showed that the proportions of strong, medium, and weak biofilm forming strains among 57 *S. aureus* isolates were 12.3, 80.7, and 7.0%, respectively. Triveni et al. (2018) reported that only 72 (38.3%) of the 188 strains of *S. aureus* were screened as biofilm producers, of which 34 (18.1%) were strong biofilm-forming strains and 38 (20.2%) were medium biofilm-forming strains. The discrepancies in the categorization of the biofilm phenotypes could result from differences in the interpretation of the results. Therefore, standardizing the methods and interpretation of biofilm formation is crucial. At the same time, *S. aureus* from different sources (human or animals) and geographical sources may also have different abilities to form biofilm. It is important to fully explain these various sources (Bissong and Ateba, 2020).

Inadequate cleaning of milking equipment leads to milk residue. These organic residues provide nutrients for the growth of bacteria during the milking process and increase the risk of microorganisms adhering to the surface and the subsequent formation of a biofilm (Alonso and Kabuki, 2019; Latorre et al., 2020). The ability of *S. aureus* biofilms to adhere to materials, such as rubber and stainless steel surfaces of appliances, and equipment at dairy farms or in dairy processing environments, has been demonstrated (Lee et al., 2014). Among them, some genes such as *icaA*, *icaD*, and *bap* play an important role in biofilm formation of *S. aureus*. The existence of these genes may represent an important mechanism of adhesion to biological or non-biological surfaces, such as milking equipment surfaces (Castelani et al., 2015; Ren et al., 2020). Therefore, further research is needed to determine the existence of known genes involved in biofilm formation, and apply them to the study of biofilm formation on dairy farm equipment and utensils.

Many different molecular typing methods have been used to compare the risk of *S. aureus*, including multiple locus variable number tandem repeat analysis, staphylococcal protein A typing, and PFGE (Roussel et al., 2015). PFGE is a highly discriminating technique to characterize the genetic diversity of different bacterial pathogens, including *S. aureus* (Tang, 2009). It is not only low cost, but does not require detailed biological information or software. PFGE remains the gold standard for classifying cooperative position staphylococci (Sarah et al., 2017). In this study, 13 pulsotypes (P1–P13) and 26 subtypes were obtained with 80% similarity among the 47 isolates according to the PFGE analysis. Among them, type P3 had the most abundant diversity of the all pulsotypes observed in Figure 1, including eight subtypes (P3-i–P3-viii) and 14 isolates, and these isolates were from two milk sources in two regions (cow milk from Shihezi and camel milk from Altay), indicating that this pulsotype was epidemic in different regions of northern Xinjiang. Sharma et al. (2017) reported that some strains spread from one region to another during milk transport, which is similar to the conclusion of this study. The PFGE results showed that the isolates were genetically diverse in terms of regional distribution and sample source.

## Conclusion

In this study, the contamination, virulence, and drug resistance characteristics, biofilm formation ability, and PFGE molecular typing of *S. aureus* isolated from different milk sources in northern Xinjiang were monitored. The results showed that the contamination level of cow and camel milk in northern Xinjiang was high, and drug resistance and biofilm formation of the strains was serious, particularly those of MRSA. Raw milk is one of the major sources of dairy production and supplementary nutrition for consumers, so its safety is important. Therefore, in order to reduce the risk of SFP caused by the intake of raw milk, especially enterotoxin-producing *S. aureus*, relevant departments should strengthen the health supervision and management of raw milk from the links of collection, transportation and sales. At the same time, these departments should strictly control the standards of using animal antibiotics to prevent public health security problems caused by excessive use and abuse of antibiotics, so as to protect the health and safety of consumers.

## Data Availability Statement

The original contributions presented in the study are included in the article/supplementary material, further inquiries can be directed to the corresponding author/s.

## Author Contributions

XK, HC, and HJ conceived and designed the experiments. XK, HC, and SH performed the experiments. XK and XW analyzed the data and wrote the manuscript. YN, BL, and HQ assisted with the sample collection and treatment. All authors contributed to the article and approved the submitted version.

## Conflict of Interest

HJ and YN are employed by School of Food Science and Technology, Shihezi University. The remaining authors declare that the research was conducted in the absence of any commercial or financial relationships that could be construed as a potential conflict of interest.

## Publisher’s Note

All claims expressed in this article are solely those of the authors and do not necessarily represent those of their affiliated organizations, or those of the publisher, the editors and the reviewers. Any product that may be evaluated in this article, or claim that may be made by its manufacturer, is not guaranteed or endorsed by the publisher.
